# Neurofibromin 1 Is a miRNA Target in Neurons

**DOI:** 10.1371/journal.pone.0046773

**Published:** 2012-10-02

**Authors:** Maria Paschou, Epaminondas Doxakis

**Affiliations:** Molecular and Cellular Neurobiology Lab, Center of Neurosciences and Social Psychiatry, Biomedical Research Foundation, Academy of Athens, Athens, Greece; University of Nebraska Medical Center, United States of America

## Abstract

Mutations of the neurofibromin 1 gene cause neurofibromatosis type 1, a disease in which learning and behavioral abnormalities are common. The disease is completely penetrant but shows variable phenotypic expression in patients. The repertoire of regulatory interactions utilized by neurons to control neurofibromin 1 expression is poorly understood. Here, we examined the contribution of microRNAs into this regulatory network. Using reporter assays, we provided evidence that miR-128 and to a lesser extent miR-137 and miR-103 reduced neurofibromin 1 reporter levels through specific binding to *Nf1* 3′-UTR. Mutations in all three predicted binding sites eliminated the reporter response. MiR-128 and miR-137, unlike miR-103 that showed a more ubiquitous expression, were predominantly expressed in brain with a distribution that resembled neurofibromin 1 expression in different tissues as well as during the course of neuronal development. In the nervous system, all three microRNAs showed highest expression in neurons and least in Schwann cells and astrocytes. Overexpression of miR-128 alone or with miR-103 and miR-137 significantly reduced endogenous neurofibromin 1 protein levels, while antisense inhibition of these microRNAs enhanced translation of endogenous neurofibromin 1 and reporter in primary cultures of hippocampal neurons. These findings revealed a significant additional mechanism by which neurofibromin 1 is regulated in neurons and implicated new candidates for the treatment of multifarious neurofibromatosis type 1 cognitive symptoms.

## Introduction

Neurofibromatosis type 1 (NF-1) is a common autosomal dominant genetic disorder that affects 1 in 3500 individuals worldwide. The disease is characterised by a spectrum of somatic and cognitive symptoms that include café au lait macules, Lisch nodules, neurofibromas and learning disabilities [1]. Individuals affected by NF-1 are heterozygous for the *Nf1* gene mutation, as homozygous mutations appear to be lethal [2]. Neurofibromin 1 (NF1), the *Nf1* gene product, is a large protein that contains a central Ras-GTPase-activating (RasGAP) domain thought to function as a negative regulator of proto-oncogene RAS and downstream effectors [3], [4], [5].

Despite the fact that NF-1 is a single-gene disease, it is presented by a variable expressivity of symptoms even within families, indicating that other modifier genes are involved in the disease process. Towards this, studies have thus far recognized ubiquitination through ETEA [6] and alternative splicing mediated by RNA binding proteins HuR and CELF as possible contributing factors [6], [7].

MicroRNAs (miRNAs) are short 17–24 nucleotides, non-coding RNAs that act to inhibit protein expression by hybridizing to complementary sequences in the 3′-UTR of closely related target transcripts. These recognition sequences demonstrate near perfect complementarity of a 7–8 nucleotides stretch at 5′end of the miRNA, termed the seed sequence, while interaction with the 3′end of miRNA requires less stringency [8]. MiRNAs confer robustness to developmental genetic programs by presumably two opposing ways. In the first case, the miRNA and target mRNAs are highly expressed in mutually exclusive tissues where the miRNA functions to block translation of the unwanted mRNAs expressed from leaky promoters. And in the second case, both the miRNA and target mRNAs are co-expressed in the same tissues where the miRNA acts as rheostat to dampen protein translation to optimal levels enabling customized expression [9], [10]. MiRNAs display a wide variety of expression patterns and many are differentially expressed during development or disease [11]. With respect to NF-1, miR-10b and miR-34a have been shown to be up- and down- regulated in Malignant Peripheral Nerve Sheath Tumors, respectively while miR-10a also participates in the regulation of NF1 levels in these tumors [12], [13].

With this in mind, we sought to further characterize the physiological regulation of NF1 expression by miRNAs. We have tested a number of predicted conserved miRNAs for NF1 regulation and identified miR-128, miR-137 and miR-103 as new regulators. These miRNAs have overlapping expression with NF1 in different tissues and are predominantly neuronal indicating they refine fluctuations of endogenous NF1 levels in the nervous system.

## Materials and Methods

### Ethics Statement

All rodent tissue was obtained in accordance with European Union (2003/65/CE) guidelines on the use of laboratory animals. Experimental protocols were approved by the Institutional Animal Care and Use Committee of BRFAA and the Veterinary Services of Attica prefecture (K/2134).

### Generation of DNA Constructs

The murine *Nf1* 3′-UTR (NM_010897, bases 8635 to 11844) was amplified by proofreading Phusion PCR (Finnzymes, Vaanta, Finland) from mouse brain DNA by using the primers 5′- CATTAGCTCGAGAGCGTTCTTTGTTTCAC -3′ and 5′-GTAATCGCGGCCGCGAGCAGATTGAATTTACATTTT -3′. The ∼3,2 kb PCR product was, then, cloned into psiCHECK-2 vector (Promega, Madison, USA) downstream from the Renilla luciferase coding sequence. Expression vectors directing the synthesis of mmu-miR-103-1 (miRBase accession no. MI0000587), mmu-miR-128-2 (miRBase accession no. MI0000726), and mmu-miR-137 (miRBase accession no. MI0000163) were prepared by introducing annealed oligonucleotides corresponding to the precursor miRNA sequences (pri-miRNA) into the pcDNA6.2-GW/EmGFP vector (Invitrogen, Carlsbad, USA). The oligonucleotide sequences were as follows:

Mmu-pri-mir-103-1 sense, 5′-

TGCTGTTCTTACTGCCCTCGGCTTCTTTACAGTGCTGCCTTGTTGCATATGGATCAAGCAGCATTGTACAGGGCTATGAAGGCATTGAGAC -3′ and

Mmu-pri-mir-103-1 antisense, 5′-

CCTGGTCTCAATGCCTTCATAGCCCTGTACAATGCTGCTTGATCCATATGCAACAAGGCAGCACTGTAAAGAAGCCGAGGGCAGTAAGAAC -3′.

Mmu-pri-mir-128-2 sense, 5′-

TGCTGCAGTGGGAAGGGGGGCCGATGCACTGTAAGAGAGTGAGTAGCAGGTCTCACAGTGAACCGGTCTCTTTCCCTACTG-3′ and

Mmu-pri-mir-128-2 antisense, 5′-

CCTGCAGTAGGGAAAGAGACCGGTTCACTGTGAGACCTGCTACTCACTCTCTTACAGTGCATCGGCCCCCCTTCCCACTGC-3′.

Mmu-pri-mir-137 sense, 5′-

TGCTGCTTCGGTGACGGGTATTCTTGGGTGGATAATACGGATTACGTTGTTATTGCTTAAGAATACGCGTAGTCGAGG -3′ and

Mmu-pri-mir-137 antisense, 5′-

CCTGCCTCGACTACGCGTATTCTTAAGCAATAACAACGTAATCCGTATTATCCACCCAAGAATACCCGTCACCGAAGC -3′.

The mmu-pri-mir-128/103 and mmu-pri-mir-128/137 dual miRNA expression plasmids were prepared by inserting mmu-pri-mir-103-1 or mmu-pri-mir-137 sequence after mmu-pri-mir-128-2 sequence in the pcDNA6.2-GW/EmGFP vector by restriction cloning. Scramble 1, supplied by Invitrogen is presumably predicted not to target any vertebrate gene. Scramble 2 was prepared by scrambling the mature sequence of mmu-miR-128-2 in the mmu-pri-mir-128-2 sequence (miRBase accession no. MI0000726) before inserting it in the pcDNA6.2-GW/EmGFP vector as previously described [14]. All vectors were checked by sequencing before use.

### Site-directed Mutagenesis

Three point mutations, one for each miRNA binding site were inserted in *Nf1* 3′-UTR as previously described [15]. Briefly, a set of four proofreading PCR reactions with mutagenized primers was carried out using the psiCHECK2-*Nf1* 3′-UTR vector as a template. The reactions were as follows (mutations are underlined):

PCR1: External wt-*Nf1* F (5′- CATTAGCTCGAGAGCGTTCTTTGTTTCAC -3′) + Mut-mir-103 R (5′- ATCAAAGAAAAGCACGATTAAACTTAGGCA -3′),

PCR2: Internal mut-mir-103 F (5′- CGTGCTTTTCTTTGATGTTTTTC -3′) + Internal mut-mir-137 R 5′- TTTTCCTGTACTTAAAGCTAAGACTCAAATG -3′),

PCR3: Internal mut-mir-137 F (5′- TTTAAGTACAGGAAAATAAGC -3′) + Internal mut-mir-128 R (5′- CGGCCAGACTTCAGTGTGACTAAATGCAA -3′),

PCR4: Internal mut-mir-128 F (5′- ACTGAAGTCTGGCCGGCCTGTG -3′) + External wt-*Nf1* R (5′- GTAATCGCGGCCGCGAGCAGATTGAATTTACATTTT -3′).

The PCR products were purified with a gel extraction kit (Qiagen, Hilden, Germany) and equal amounts were PCR-assembled, two fragments at a time, using corresponding flanking primers. The cycling conditions were as follows: five cycles with twice as long extension times and no primers -to allow assembly of full fragment-, followed by 15 cycles of proofreading PCR to amplify the assembled fragment. The mutagenized final PCR product was then cloned into the psiCHECK-2 plasmid and sequenced.

### Lentivirus Production

The vectors PLL3.7/EmGFP-pri-mir-128 and PLL3.7/EmGFP-pri-mir-128/103, and PLL3.7/EmGFP-pri-mir-128/137 were constructed by replacing the U6 promoter and GFP sequences between WRE and FLAP of the PLL3.7 vector (kindly provided by Ford E, BRFAA) with the EmGFP-pri-mir cassettes from the pcDNA6.2-GW/EmGFP-pri-mir-128, pcDNA6.2-GW/EmGFP-pri-mir-128/103 and pcDNA6.2-GW/EmGFP-pri-mir-128/137 plasmids, respectively. Viral particles were prepared by co-transfection of HEK293T cells with PLL3.7/EmGFP-pri-mirs, helper pCMV-dR8.91 and pMD2.G plasmids at 16∶7: 7 µg DNA ratios per 10 cm plate by using the calcium phosphate method. After 48 hours, the supernatants were spun at 2,000 rpm for 6 minutes, filtered at 0.45 µm pore size, and spun at 26,000 rpm (Sorvall TH-641 rotor) for 1 hour 30 minutes before pellets were resuspended in 100 µl of 1% bovine serum albumin. Lentiviral titers were determined by infecting HEK293 cells with serial dilutions of the viral stock for 48 hours and followed by fluorescent-activated cytometric sorting (BD FACSAria sorter, Franklin Lakes, USA). Titers were approximately 5×10^7^ infectious units per ml.

### Cell-line Culture and Transfection

HEK293 cells were maintained in low-glucose DMEM (GIBCO-Invitrogen) supplemented with 10% fetal bovine serum (FBS) (Biowest, Nuaillé, France). For luciferase reporter assay, HEK293 cells were transfected a day after plating by using Lipofectamine 2000 according to the manufacturer’s instructions (Invitrogen). To ensure that transfection efficiencies and NF1 reporter load onto cells were uniform across conditions both luciferase psiCHECK-2 construct and Lipofectamine 2000 reagent were prepared as master mix before aliquoting into tubes containing the different miRNA plasmids. Transfection efficiencies were at 60–80% at 48 hours as indicated by GFP. For western blotting assay, HEK293 cells were transfected at plating by using Lipofectamine 2000 according to the manufacturer’s instructions. Six hours later, cells were infected with lentiviral vectors to boost transfection efficiency to near 90% by 48 hours.

**Figure 1:**
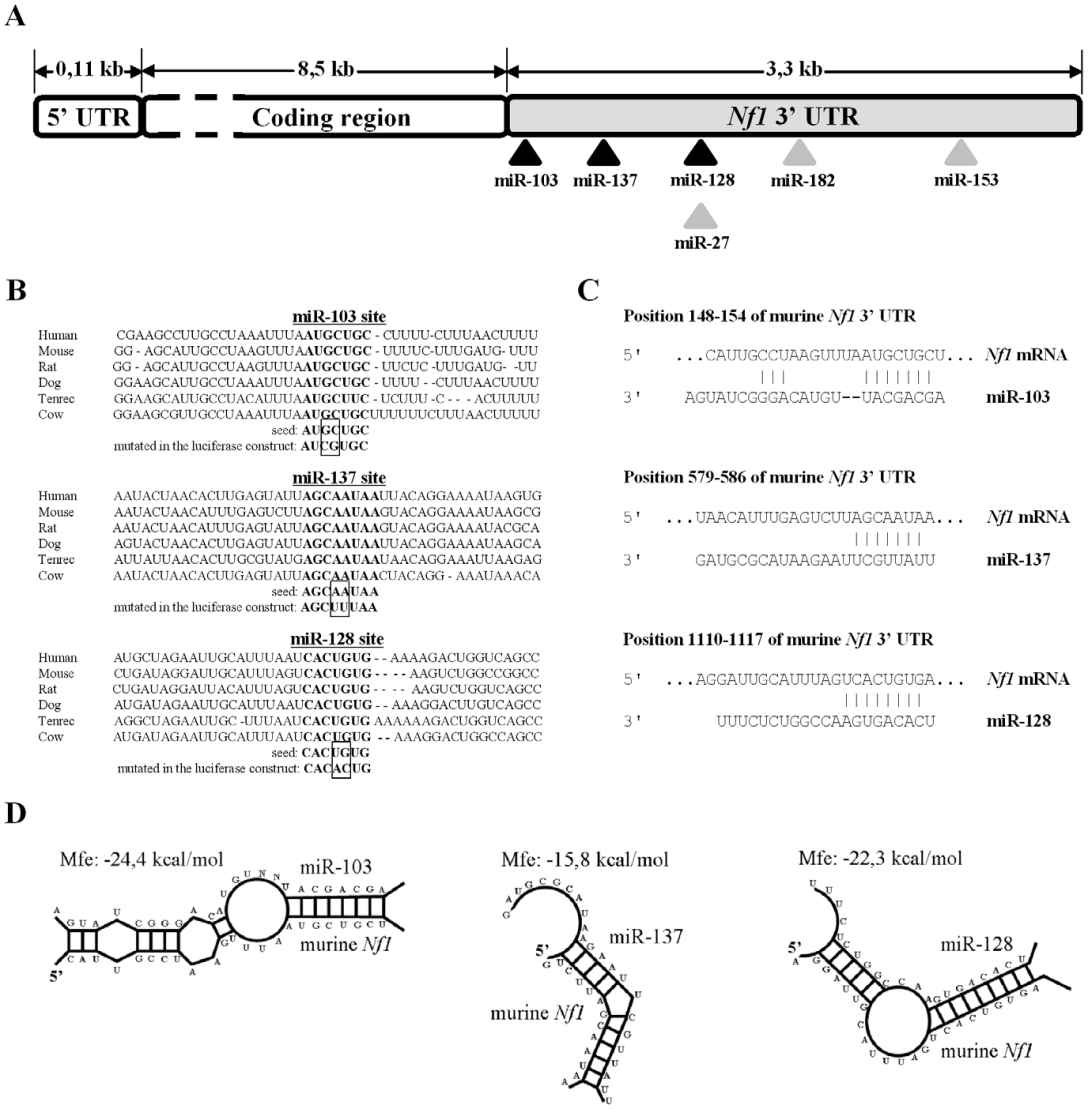
MiRNA binding sites in Nf1 3′-UTR. (**A**) Relative position of conserved miR-103, miR-137, miR-27, miR-128, miR-182 and miR-153 sites in *Nf1* 3′-UTR. (**B**) Sequence conservation of miR-103, miR-137 and miR-128 binding sites. (**C**) Alignment of mature miR-103, miR-137 and miR-128 sequences with *Nf1* transcript. (**D**) Predicted hybridization of miRNAs and *Nf1* transcript using the RNAhybrid algorithm [36]. The minimum free energy required for the hybridization is indicated.

### Neuron Culture and Transfection

Dissociated, embryonic day 16–17 murine hippocampal or cortical neurons (>95% pure), were grown in Neurobasal medium with B-27 supplement (GIBCO, Invitrogen) and Glutamax (GIBCO, Invitrogen) in poly-L-lysine (SIGMA, St-Louis, USA) coated borosilicate glass coverslips (VWR, Radnor, USA) in the absence of trophic factors [16]. For *Nf1* mRNA analysis, ∼20 hours after plating, neurons were infected with miR-128 lentiviral vectors and assayed 48 hours later by RT-PCR. For NF1 protein or reporter analysis, neurons were transfected using Lipofectamine 2000 reagent straight after dissociation with the a) miRNA expression plasmids or b) the inhibitor 2′-O-methyl oligonucleotides plus luciferase psiCHECK-2 or pmaxGFP constructs (Lonza, Basel, Switzerland) according to the manufacturer’s instructions (Invitrogen) and assayed ∼40 hours later by immunocytochemistry or Dual-Luciferase reporter assay. The antisense (as) 2′-O-methyl RNA inhibitor sequences were: for scramble, CAGUACUUUUGUGUAGUACAA; for as-mmu-miR-103, UCAUAGCCCUGUACAAUGCUGCU; for as-mmu-miR-137, CUACGCGUAUUCUUAAGCAAUAA, and for as-mmu-miR-128, AAAGAGACCGGUUCACUGUGA (GenePharma, Shanghai, China). For co-transfection conditions, neurons received half the amount of 2′-O-methyl oligonucleotides per miRNA in order to have comparable levels of transfected oligonucleotides to control and mmu-miR-128 conditions.

### Astrocyte Culture

Embryonic day 17 murine cortical tissues, free of meninges, were treated with 0.05% trypsin solution (Worthington, Lakewood, USA) for 5 min at 37°C, then dissociated using a fire-polished Pasteur glass pipette and plated in tissue culture dishes (Greiner, Kremsmünster, Austria) containing RPMI (Biosera, East Sussex, UK) and 10% FBS (Biowest). After approximately one week of culture, astrocytes were trypsinized, dispersed in RPMI plus 10% FBS and re-plated at 1∶3 the original density. They were harvested for mirVana RNA isolation after becoming confluent again.

**Figure 2:**
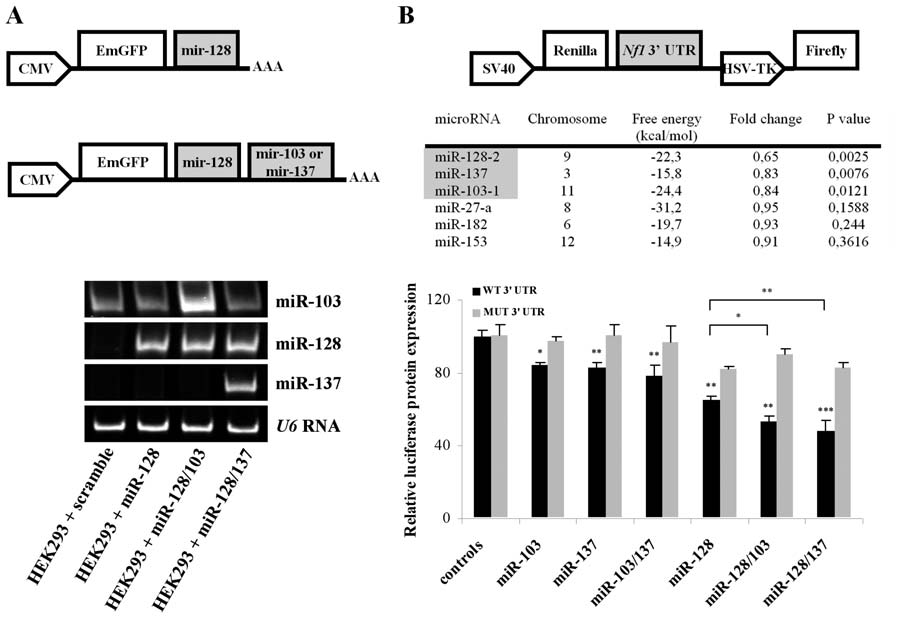
MiR-103, miR-128 and miR-137 directly reduce NF1 reporter protein expression. (**A**) Schematic of miRNA plasmids and their expression levels in HEK293 cells. Representative gel showing the products of RT-PCR reactions amplified with primers specific for mature miR-103, miR-128, and miR-137 in HEK293 cells transfected with scramble or miR-128 vectors. The amount of starting template for each condition was equilibrated relative to *U6* RNA. Cycles were falling within the linear range of amplification for each primer pair. (**B**) Schematic of *Nf1* 3′-UTR reporter construct and miRNAs tested for *Nf1* 3′-UTR regulation of expression. HEK293 cells were co-transfected with both the reporter gene (0.2 µg/reaction) and miRNA expression vectors (pri-mir-103, pri-mir-128, pri-mir-137, pri-mir-27, pri-mir-182 and pri-mir-153) (0.8 µg/reaction) and luciferase activity was measured 48 hours later. The fold change from control values as well as the *P*-value for each miRNA condition is presented in the table. These assays demonstrated that only miR-103, miR-128 and miR-137 significantly reduce NF1 reporter protein levels. Subsequently, mutagenesis of the predicted miR-103, miR-128 and miR-137 binding sites alleviated this inhibitory effect indicating that miR-103, miR-128 and miR-137 directly suppress *Nf1* expression by targeting the identified seed regions in the *Nf1* 3′-UTR. The average value of three single (scramble 1, scramble 2, miR-218) and two double (miR-101/181, miR-218/377) miRNA constructs expressing miRNA predicted not to bind *Nf1* 3′-UTR were used as controls for the single or double expression constructs, respectively. Data show the mean ± s.e.m from 6 independent transfections (*, P<0.05; **, P<0.01).

### Schwann Cell Culture

Schwann cells were cultured from bilaterally excised sciatic nerves of postnatal day 0 mice. The cells were dissociated with 0.125% trypsin and 0.2% collagenase (Worthington) solutions for 40 min at 37°C, then triturated through a fire-polished Pasteur pipette and plated in poly-L-lysine coated (SIGMA) tissue culture dishes. Cells were grown in low-glucose DMEM supplemented with 10% FBS. They were harvested for mirVana RNA isolation after becoming confluent.

### Measurement of miR-103, miR-137, miR-128, Nf1 and U6 RNAs

A semi-quantitative RT/PCR assay was used to compare the levels of miR-103, miR-137, miR-128, *Nf1* and *U6* mRNAs in the various cell types and tissues. Total RNA was isolated using the mirVana miRNA isolation kit (Ambion, Austin, USA) and recovered in DEPC-treated H_2_O. 0.5 µg RNA was reverse transcribed for 1 hour at 42°C with superscript II enzyme (Invitrogen) in reaction containing the manufacturer’s buffer and DTT supplemented with 0.5 mM dNTPs (Promega), 10 µM random hexanucleotides (Amersham/GE Healthcare, Buckinghamshire, UK) and 2.5 pmoles of gene-specific primers for mature hs/mmu-miR-103 (5′-CATGATCAGCTGGGCCAAGATCATAGCC-3′), hs/mmu-miR-137 (5′- CATGATCAGCTGGGCCAAGACTACGCGT-3′), and hs/mmu-miR-128 (5′- CATGATCAGCTGGGCCAAGAAAAGAGAC-3′). These primers included an extension sequence (underlined) to facilitate subsequent amplification and monitoring by PCR {modified from [17], [18]}.

**Figure 3:**
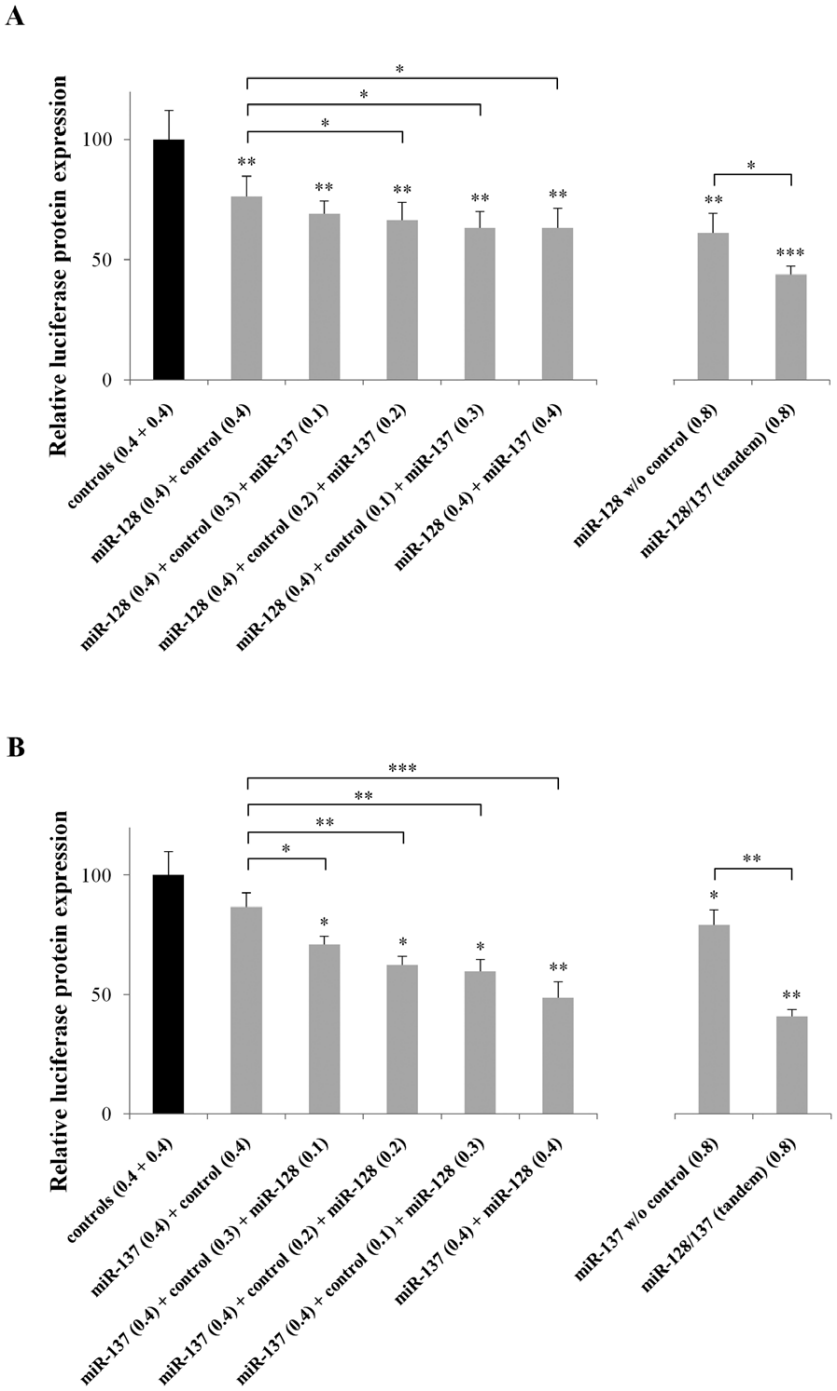
MiR-128 and miR-137 synergize to reduce NF1 reporter protein expression. HEK293 cells were co-transfected with both the reporter gene (0.2 µg/reaction) and variable concentrations of pri-mir-128 and pri-mir-137 expression vectors. Luciferase activity was measured 48 hours later. (**A**) pri-mir-128 plasmid concentration was kept at 0.4 µg/reaction while pri-mir-137 concentration varied from 0.1–0.4 µg/reaction. (**B**) pri-mir-137 plasmid concentration was kept at 0.4 µg/reaction while pri-mir-128 concentration varied from 0.1–0.4 µg/reaction. Scramble 2 plasmid was supplemented in some of the reactions to achieve final miRNA plasmid concentration of 0.8 µg/reaction. These assays demonstrated that miR-128 and miR-137 synergistically reduce NF1 reporter expression when levels of the less abundant miRNA in the reaction are 50% or higher of the more abundant miRNA. The average value of two single miRNA expression plasmids (combination of scramble 2 plus pri-mir-9 or pri-mir-181 or pri-mir-218) predicted not to bind *Nf1* 3′-UTR were used as controls. Data show the mean ± s.e.m from 4 independent transfections (*, P<0.05; **, P<0.01, ***, P<0.001).

Following reverse transcription, duplicate measurements of 2 µl of 2.5 times diluted in H_2_O cDNA were made in 10 µl PCR reactions containing 1×buffer, 0.5–1.5 mM MgCl_2_, 0.2 mM dNTPs, 4 pmoles primers, and 1.25 units DreamTaq (Fermentas, Burlington, Canada). The forward primers were as follows: hs/mmu-miR-103 F, 5′-TGTGTGAGCAGCATTGTACAGG-3′; hs/mmu-miR-137 F, 5′-GCCGGGTTATTGCTTAAGAATAC-3′; hs/mmu-miR-128 F, 5′-TCTGGGTCACAGTGAACCGGT-3′; mmu-*Nf1* F, 5′-GTGGCCCCTACAAATCATTCTC-3′; hs-*Nf1* F, 5′-GCTTTCGTATAAGCCCTCACAA-3′; and hs/mmu-*U6* F, 5′-CGCTTCGGCAGCACATATAC-3′. The reverse primers were as follows: Universal reverse for miR-103, miR-137 and miR-128, 5′-CATGATCAGCTGGGCCAAGA-3′; mmu-*Nf1* R, 5′-TGCTTTCCGTAAACTGTCCAG-3′; hs-*Nf1* R, 5′-GCGGAATTGGTGATGATTCGATG-3′and hs/mmu-*U6* R, 5′-TTCACGAATTTGCGTGTCAT-3′. cDNAs were amplified by cycling at 95°C for 20 sec, followed by 20 sec at 55°C, followed by 20 sec at 72°C. The reaction was then completed with a 10 min extension at 72°C. Cycles were falling within the linear range of amplification for each primer pair. These were 24–26 cycles for *U6* RNA and 27–30 cycles for miR-103, miR-128, miR-137 and *Nf1* mRNAs. The PCR products were, next, separated on 8% non-denaturing polyacrylamide gels. The gels were, subsequently, stained with SyberGold (Invitrogen) and images were captured with the Dolphin gel documentation system (Wealtech, Sparks, USA).

### Luciferase Reporter Assay

Luciferase assays were performed 40–48 hours after transfection with the Dual-Luciferase reporter kit (Promega) and measured with the Lumat LB9507 luminometer (Berthold Technologies, Bad Wildbad, Germany). Changes in expression of Renilla luciferase (target) were calculated relative to firefly luciferase (internal control).

### Western Blotting

Western blotting was used to assay NF1 protein levels in HEK293 cells transduced with the appropriate miRNA expression vectors. Two days later, whole-cell protein extracts were harvested with Urea lysis buffer (8 M urea, 0.5% SDS, 1 mM Na_3_VO_4_), sonicated and boiled for 5 min. For Western blotting, 10–30 µg of each protein extract was loaded onto denatured 7.5% polyacrylamide gel and transferred to Protran® nitrocellulose membrane (Whatman, Kent, UK). The membrane was incubated in 5% bovine serum albumin (BSA) for 1 hour followed by overnight incubation with polyclonal NF1 (sc-67, Santa Cruz Biotechnology, Santa Cruz, California, USA) and monoclonal β-tubulin (clone D66, SIGMA) antibodies. Next day, the membrane was incubated for 1 hour with anti-rabbit (sc-2054, Santa Cruz Biotechnology) for NF1 and anti-mouse (sc-2055, Santa Cruz Biotechnology) for β-tubulin secondary antibodies conjugated with horse radish peroxidase (HRP). HRP signals were, then, detected using the Western Lighting Plus ECL reagents (PerkinElmer, Waltham, USA) according to the manufacturer’s instructions.

**Figure 4:**
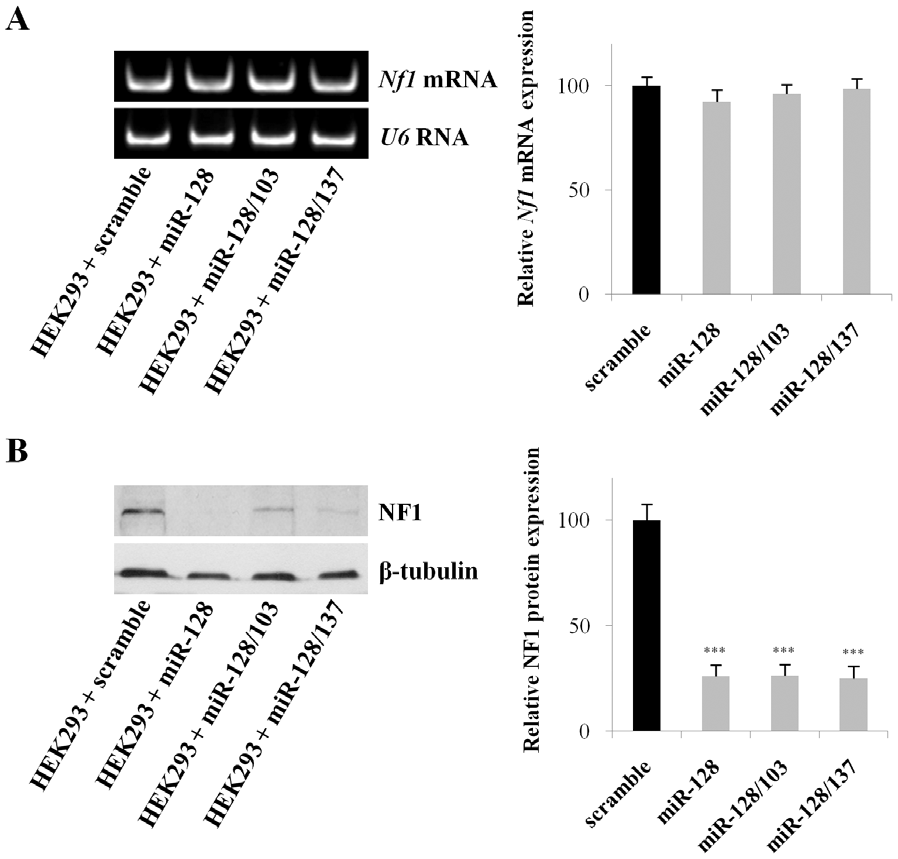
MiR-103, miR-128 and miR-137 reduce endogenous NF1 protein but not mRNA expression. (**A**) Representative gel of the RT-PCR amplification of *Nf1* mRNA in HEK293 cells transfected with scramble or miR-128 vectors for 48 hours. The amount of starting template for each condition was equilibrated relative to *U6* RNA. Cycles were falling within the linear range of amplification for each primer pair. (**B**) Representative Western Blot analysis demonstrating that miR-128, miR-128/103, and miR-128/137 reduce endogenous NF1 protein levels in human cells. HEK293 cells were transfected with miR-128 constructs for 48 hours. 15 µg of whole-cell lysate was then loaded in each lane. B-tubulin was used as an internal control for loading. Data show the mean ± s.e.m from 4 (for RT-PCR) and 8 (for Western Blot) independent experiments (***, P<0.001). B

### Immunocytochemistry and Densitometry

Neurons were fixed 48 hours after transfection with freshly-made 4% (w/v) paraformaldehyde solution (SIGMA) and briefly permeabilised with 0.4% Triton X-100 (SIGMA). Cells were subsequently blocked with 5% of secondary serum (normal goat serum, Santa Cruz Biotechnology) and BSA in 1∶1 ratio and incubated overnight at 4°C with primary NF1 antibody. Next day, cells were incubated for 1 hour with goat anti-rabbit Alexa Fluor 546 secondary antibody (Molecular Probes/Invitrogen) and mounted with Vectashield (Molecular Probes/Invitrogen) media. Images were taken with a Leica DMRA2 upright microscope using a 60×objective. NF1 levels in scrambles, miR-128, miR-128/103 or miR-128/137 transfected cells were estimated by comparing the average intensity of five neurons with brightest Alexa Fluor 546 signal in each field with the miRNA transfected, GFP-positive, neuron in the same field (80 fields in total per miRNA condition per experiment) using ImageJ [19]. Duplicate transfections were set up for the each condition, and the data were compiled from six separate experiments. Statistical significance was assessed using the student's paired *t*-test (two-tailed distribution).

**Figure 5:**
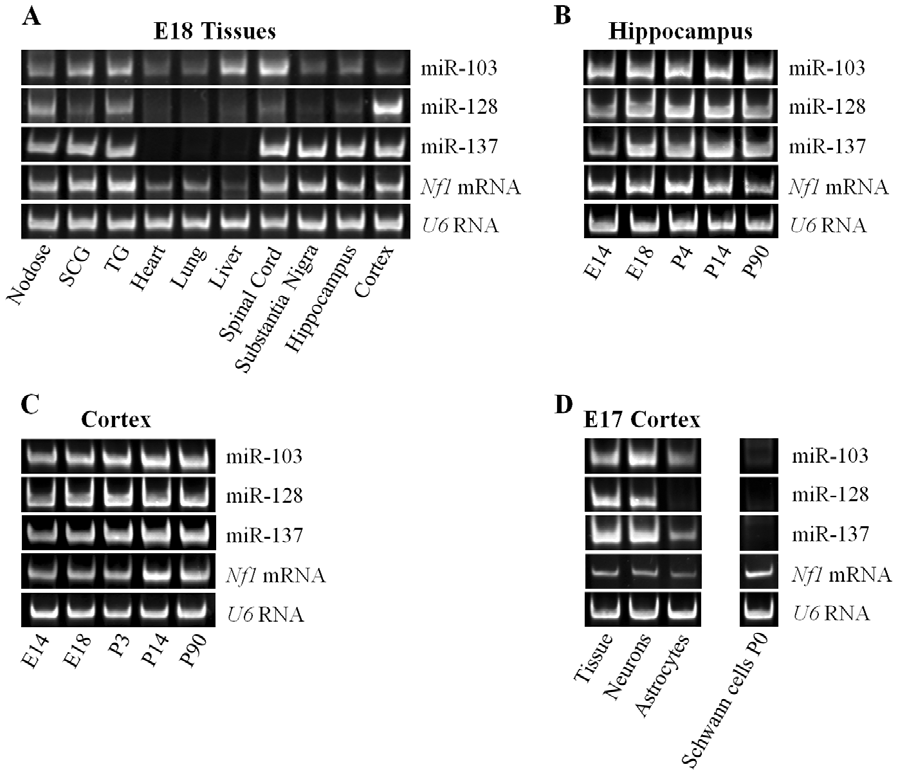
MiR-103, miR-128, miR-137 and Nf1 mRNA are co-expressed in the nervous system. Representative gels of the RT-PCR amplification products of miR-103, miR-128, miR-137, and *NF1* mRNA levels in: (**A**) Different murine tissues of embryonic day 18 animals; (**B**) Hippocampus of different ages; (**C**) Cortex of different ages; and (**D**) Different neural cell types. The amount of starting template for each condition was normalized to *U6* RNA. The number of PCR cycles for each miRNA RT-PCR assay is different between gels. Cycles were falling within the linear range of amplification for each primer pair. SCG, superior cervical ganglion; TG, trigeminal ganglion; E, embryonic day; P, postnatal day.

## Results

### Multiple miRNA Target Sites are Predicted in Nf1 3′-UTR

To identify putative miRNA target sites located within the 3.5 kb 3′-UTR of *Nf1* transcript, predictions from four different algorithms were compiled and compared. These were TargetScan [20], miRanda [21] Pictar [22] and Diana microT [23]. Several putative target sites, which were conserved at orthologous positions across multiple vertebrate species, were identified by all four software (Fig. 1*A*). They included sites for miR-27, miR-103, miR-128, miR-137, miR-153, and miR-182. Sequence conservation and bioinformatics prediction for three of these sites is shown in Fig.1*B and C*. The miR-103 predicted site (seed sequence position 148–154) has a minimum free energy of −24.4 kcal/mol, the miR-137 site (seed sequence position 579–586) has a minimum free energy of −15.8 kcal/mol and the miR-128 site (seed sequence position 1110–1117) has a minimum free energy of −22.3 kcal/mol. The predicted sites were all flanked by a large number of AU residues indicating that *Nf1* mRNA sequence may facilitate miRNA binding at these sites (Fig. 1*B*) [20].

**Figure 6:**
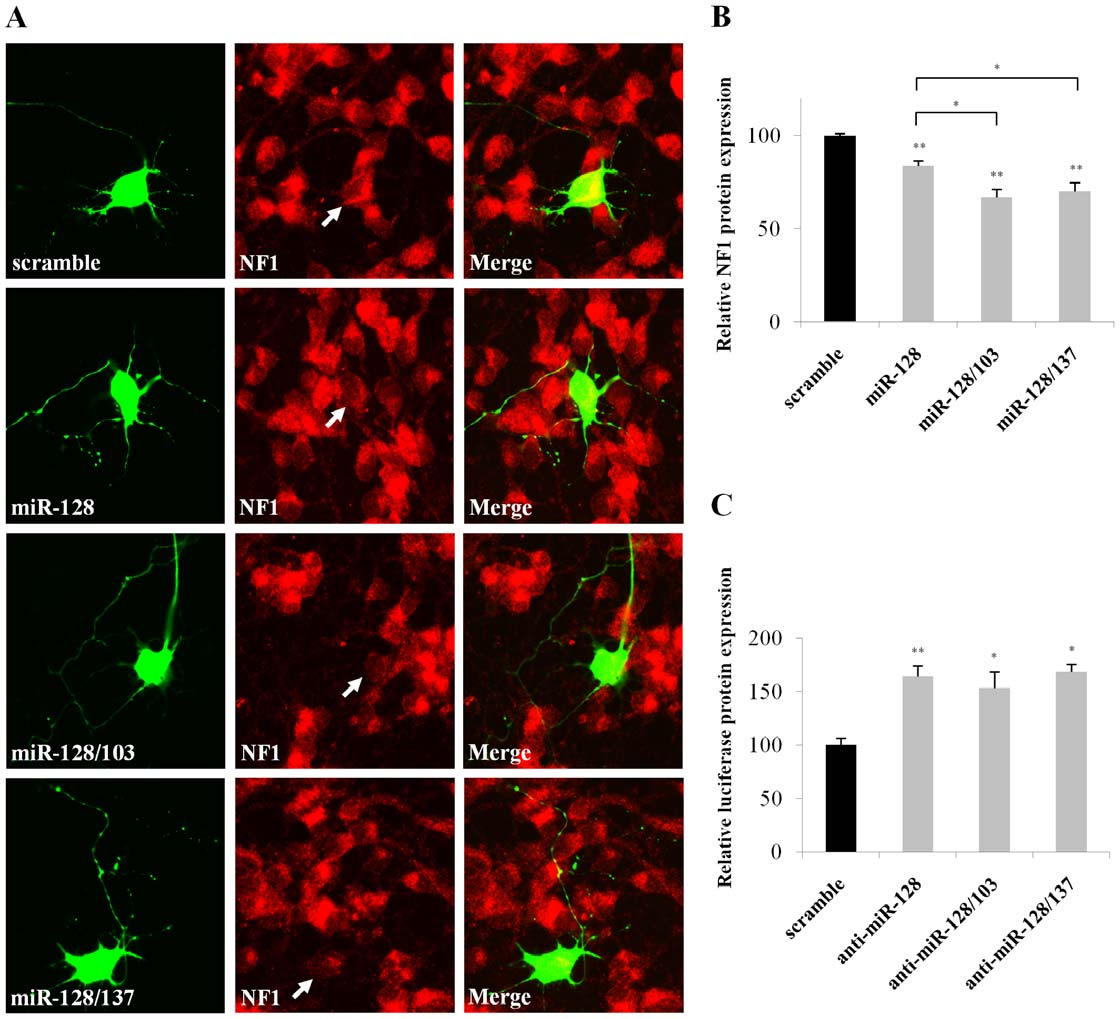
MiR-128 alone or together with miR-103 or miR-137 reduces endogenous NF1 protein levels in neurons. (**A**) Representative images of transfected hippocampal neurons (green) stained with NF1 antibody (red). Merged images are shown in the third column of the panel. E16 murine hippocampal neurons were transfected with scramble or pri-mir-128 plasmids immediately after plating by using Lipofectamine 2000. Immunocytochemistry was carried out 40 hours after transfection. (**B**) Average decrease in NF1 protein levels. 80 neurons were analyzed per experiment. Data show the mean ± s.e.m from 6 independent experiments (*, P<0.05; **, P<0.01). (**C**) Freshly dissociated hippocampal neurons were transfected with *Nf1* reporter vector plus antisense 2′-O-methyl inhibitors (miR-128 alone or together with miR-103 or miR-137) and assayed 48 hours later. Data show the mean ± s.e.m from 4 independent experiments (*, P<0.05).

### MiR-103, miR-128 and miR-137 Functionally Interact with Nf1 3′-UTR to Reduce Protein Levels

To test whether any of the six miRNAs mediate regulatory effects on NF1 translation via *Nf1* 3′-UTR, expression plasmids, with the pri-miRNA sequences of the miRNAs inserted in the 3′UTR of Emerald GFP, were constructed (Fig. 2*A*). In addition, a reporter plasmid containing the complete *Nf1* 3′-UTR, inserted downstream of Renilla luciferase gene, was generated. This reporter plasmid, also, contained a downstream firefly luciferase gene that was used as an internal control to normalize for differences in handling and transfection efficiencies (Fig. 2*B*). For the assay, the reporter was co-transfected with the miRNA expression plasmids into HEK293 cells and luciferase reporter expression was assessed 48 hours post-transfection. HEK293 cells were used for these experiments because they express low levels of these miRNAs and are transfected with high efficiency (Fig. 2*A* and data not shown). MiR-103, miR-128, miR-137, miR-103/137, miR-128/103, and miR-128/137 (the latter three are plasmids that express two miRNAs in tandem) produced a 15.7% (P<0.05), 34.9% (P<0.01), 17% (P<0.01), 21.3% (P<0.01), 46.6% (P<0.01), and 51.7% (P<0.001) decrease in luciferase activity compared to control vectors, respectively. In contrast, miR-27, miR-182 and miR-153 did not, significantly, reduce reporter expression (Fig. 2*B*).

To confirm that this effect was mediated via the predicted sites in *Nf1* 3′-UTR, mutations in the seed sequences of miR-103, miR-137, and miR-128 were introduced in the luciferase reporter construct (Fig. 1*B*). The mutagenized reporter was then co-transfected with the miRNA expression plasmids in HEK293 cells and Renilla expression was compared to wild-type reporter. Mutations on these sites abolished the inhibitory effect of miR-103, miR-137, and miR-128 (Fig. 2*B*). These findings indicated that miR-103, miR-137, and miR-128 interact, directly, with the specified regions in *Nf1* 3′-UTR to inhibit translation from the chimaeric transcript and their effect is additive.

To determine the stoichiometric ratio at which the miRNAs were having an additive effect on reducing NF1 levels, the two most effective miRNAs, miR-128 and miR-137, were combined in different ratios (4∶1, 4∶2, 4∶3, 4∶4) and their influence on luciferase reporter expression was assessed 48 hours post-transfection. For both miRNAs, a significant additive effect was only observed when levels of the less abundant miRNA were 50% or higher of the more abundant miRNA (Figs. 3*A and B*). These findings indicated that adequate levels of both miRNAs are a prerequisite for efficient co-regulation of expression.

To examine whether miR-128, the most potent of all miRNAs, alone or together with miR-103 or miR-137, reduce endogenous NF1 levels, HEK293 cells were transfected with the miR-128 expression plasmids and *Nf1* mRNA and protein levels were assessed 48 hours later. RT-PCR from RNA extracts using *Nf1* specific primers revealed that *Nf1* mRNA expression was not, significantly, regulated by miR-128 or miR-128 double constructs (Fig. 4*A*). On the other hand, Western blots from whole protein extracts revealed a significant reduction in NF1 levels with all three constructs; miR-128, miR-128/103 and miR-137/128 produced a 74% (P<0.001), 74%, (P<0.001), and 75% (P<0.001) reduction in endogenous NF1 protein levels, respectively (Fig. 4*B*). The data indicated that these miRNAs induced translation inhibition of endogenous NF1 expression without affecting mRNA levels. Further, they showed no synergy in regulating NF1 protein levels at this cell population.

### MiR-103, miR-128, miR-137 and Nf1 mRNA are Co-expressed in Neurons

To begin characterizing the physiological interaction between miR-103, miR-137, miR-128 and *Nf1*, the levels of mature miR-103, miR-137, and miR-128 as well as *Nf1* mRNA were compared in different tissues of embryonic day 18 mice. To equate the RNA levels of these tissues, the RNA levels for the ubiquitously expressed *U6* RNA were also measured. Fig. 5*A* shows that miR-103 was ubiquitously expressed in E18 tissues with highest levels in spinal cord, liver, superior cervical (SCG) and trigeminal (TG) ganglion. MiR-128 was, predominantly, expressed in neural tissues with highest levels in cortex, nodose, trigeminal and SCG ganglia, and least in spinal cord, substantia nigra and hippocampus. MiR-137 was, exclusively, expressed in the nervous system with comparable levels in all neural tissues while *Nf1* mRNA was expressed ubiquitously in all E18 tissues tested, with highest levels in the nervous system. These patterns of expression indicated that NF1 is most likely regulated by miR-103, miR-137, and miR-128 in the nervous system where levels were highest for these interactors.

Further, to determine if miR-103, miR-137, and miR-128 expression correlates with *Nf1* mRNA expression throughout development, the levels of mature miR-103, miR-137, miR-128 and *Nf1* mRNA were also profiled in cortex and hippocampus at different ages. Figs. 5*B and C* show that the levels of miR-103, miR-137, and miR-128 corresponded overall to the expression of *Nf1* mRNA, with lower levels early in embryonic development that peak in the first two weeks of postnatal development.

Since, neural tissues are a mixed population of neurons and glial cells that include astrocytes in central nervous system and Schwann cells in peripheral nervous system, the levels of mature miR-103, miR-137, miR-128 and *Nf1* mRNA were also quantified in these different cell types. Fig. 5*D* shows that the levels of miR-103, miR-137, miR-128 and *Nf1* mRNA were significantly higher in cultures of cortical neurons than astrocytes. With respect to Schwann cell cultures, miR-103, miR-137, and miR-128 showed little or no expression while *Nf1* mRNA had highest levels. Taking all together, these results indicated that miR-103, miR-137, and miR-128 are likely regulating NF1 levels in neurons only. Moreover, since the three miRNAs and *Nf1* mRNA had similar rather than complementary distribution, it suggests that miR-103, miR-137, and miR-128 fine-tune, rather than block, NF1 protein levels in the nervous system.

### MiR-103, miR-128 and miR-137 Modulate Endogenous NF1 Levels in Neurons

Based on the initial cell-line findings and the expression data, it was hypothesized that changes in miR-103, miR-137, and miR-128 levels would, also, impact endogenous NF1 protein but not mRNA levels in primary neurons. To test this, hippocampal neurons were transfected with miR-128 alone or in combination with miR-103 and miR-137 vectors and *Nf1* mRNA and protein levels were assessed ∼48 hours later. Hippocampal neurons were selected for this experiment because they are a rather homogeneous cell population that provides enough large-size neurons for manipulation and is directly implicated in neurofibromatosis type I [24]. *Nf1* mRNA levels were assessed by RT-PCR after infecting hippocampal neurons with the pri-mir-128 lentiviral vectors. Similar to the results obtained with HEK293 cell manipulations, *Nf1* mRNA levels remained unaltered (data not shown). NF1 protein levels were assayed in GFP-positive neurons, transfected with pri-mir-128 plasmids, by immunofluorescence and quantitated by ImageJ densitometry. An average decrease of 16.1% (P<0.01), 33.1% (P<0.05), and 30% (P<0.05) in endogenous NF1 protein levels was measured with miR-128, miR-128/103, and miR-128/137 plasmid overexpression, respectively (Figs. *6A and B*). These findings indicated that a) miR-103, miR-137, and miR-128 function synergistically to regulate endogenous NF1 protein but not mRNA levels in neurons, and b) endogenous miR-103, miR-137, and miR-128 expression levels are not saturated for NF1 regulation of expression in neurons, thus, allowing a controlled amount of *Nf1* mRNA to be translated in accordance with the tuning hypothesis.

Next, the ability of endogenously expressed miR-103, miR-137, and miR-128 to regulate the levels of the reporter vector bearing the *Nf1* 3′-UTR was evaluated in the same cultures of hippocampal neurons. Antisense 2′-O-methyl as-miR-128, as-miR-128 plus as-miR-103 or as-miR-128 plus as-miR-137 inhibitors were transfected together with the *Nf1* reporter plasmid in freshly dissociated hippocampal neurons and luciferase activity was assayed 48 hours later. A 64% (P<0.01), 54% (P<0.05), and 68% (P<0.05) increase in luciferase levels were measured with antisense as-miR-128, as-miR-128/103 and as-miR-128/137 2′-O-methyl inhibitors, respectively (Fig. 6*C*) indicating that endogenous miR-128, miR-103 or miR-137 bind to mRNAs bearing *Nf1* 3′-UTR and block translation. Similar results were obtained when the inhibitors were transfected together with a GFP construct to mark transfected hippocampal neurons and NF1 protein levels were assayed by immunofluorescence and quantitated by ImageJ densitometry. An average increase of 13.4% (P<0.05, n = 3), 13.5% (P<0.05, n = 3), and 12.2% (P<0.05, n = 3) in endogenous NF1 protein levels was measured in these neurons with miR-128, miR-128/103, and miR-128/137 inhibition, respectively (data not shown). The later effects were less pronounced than those obtained using the *Nf1* reporter maybe due to detection of NF1 protein produced before transfection, the differential distribution of NF1 inside cells (nucleocytoplasmic shuttling), and/or other factors. An additive effect for the double antisense miRNA transfections was also not observed, maybe as a result of reduced as-miR-128 delivered in neurons since for the double transfections as-miR-128 amount was halved in order to have comparable amount of 2′-O-methyl oligonucleotides loaded in all conditions.

## Discussion

This study has revealed a previously unknown mechanism by which NF1 levels are regulated in the nervous system. Specifically, three miRNAs, miR-128, miR-103 and miR-137 have been shown to bind directly to the 3′-UTR of *Nf1* mRNA and significantly reduce its protein levels. This discovery has been based on: 1) conservation analysis of the recognition elements in the 3′-UTR of NF1 that show they are conserved throughout vertebrate species; 2) *in vitro* experiments in which the ‘seed’ regions have been mutagenized that resulted in the abrogation of protein degradation of a chimaeric luciferase reporter bearing the *Nf1* 3′-UTR; 3) gain-of-function experiments in which overexpression of miR-128, miR-128/103 or miR-128/137 resulted in down-regulation of endogenous NF1 protein levels in both cell-lines and hippocampal neurons; and 4) loss-of-function experiments in which inhibition of endogenous miR-128, miR-103 and miR-137 by antisense oligonucleotides promoted the expression of NF1 reporter and native protein in hippocampal neurons.

We have, also, presented data on miR-103, miR-128, miR-137 and *Nf1* mRNA levels across different tissues as well as different neuron types and glial cells. We found that miR-137 and miR-128, like NF1, had a neural distribution while miR-103 had a more ubiquitous tissue distribution. With respect to development, all three miRNAs and *Nf1* mRNA had their levels upregulated from their lowest levels at embryonic day 14 to peak levels around two weeks into postnatal development. Further, all three miRNAs were expressed highest in neurons and least in Schwann cells. We, also, found some lower expression of miR-103 and miR-137 in astrocytes. By considering the two opposing physiological means of miRNA function, these findings suggest that miR-103, miR-128, and miR-137 play a role in modulating, rather than blocking, NF1 expression in the nervous system and, in particular, neurons.

NF1 possesses several biochemical domains of which the RasGAP domain is best characterized for its ability to negatively regulate RAS signaling. RAS, a GTPase protein, tethered to the cell membrane, plays a central role in transmitting the extracellular signals from receptor tyrosine kinases, including growth factor receptors, to the nucleus. In cells, loss of NF1, leads to the constitutive activation of RAS signaling and decoupling from extracellular messages. As a result, patients with NF-1 develop malignancies and complex cognitive symptoms that include learning disabilities, attention deficit disorder and motor coordination problems. Two different splice variants, type I and II, of the NF1 RasGAP domain exist. The type I variant lacks exon 23a and it is ten times more active RasGAP than type II. Further, type I is predominantly expressed in neurons while type II in glia [25], [26]. Based on these data, a likely role of the neuronal miRNAs maybe to tightly control the ‘more active’ NF1 in neurons and, thus, facilitate a precise fine-tuning of RAS activity in these cells.

Beyond miR-103, miR-128, and miR-137 another miRNA is capable of regulating NF1 expression. MiR-10b, an oncogenic miRNA, has recently been shown to regulate NF1 expression in non-neuronal cells [13]. Interestingly, miR-10b and NF1 are expressed in mutually exclusive cells indicating that miR-10b functions to block expression of NF1. In conjunction with our data, this indicates that different cell types of the body utilize different miRNA modes of action to regulate NF1 expression.

MiR-103, miR-128, and miR-137 have all ascribed functions apart from regulating NF1 expression. MiR-103 is downregulated in neuropathic animals while intrathecal applications relieve pain [27]; it has, also, been shown to negatively regulate BDNF, a prominent neurotrophic factor involved in neuronal ERK signaling and implicated in a plethora of neuropsychiatric diseases [28], [29]. MiR-128 regulates the formation of fear-extinction memory [30] and reduces AKT signaling by repressing receptor tyrosine kinases EGFR and PDGFα [31]; it is, also, deregulated in Alzheimer’s disease [32]. MiR-137 is enriched in synaptic fractions [32], [33] and has been shown to regulate neuronal maturation and dendritic morphogenesis [34] while mutations in the gene have been associated with cognitive impairment [35]. All, these findings indicate that miR-103, miR-128 and miR-137 control a network of proteins previously found to be associated with NF1 signaling. Noting the spatial localization of mir-137 at synapses, it is possible that NF1 is, also, spatiotemporally regulated in neurons to control synaptic transmission.

In conclusion, this study provided strong evidence that miR-128, miR-103 and miR-137 regulate NF1 levels in neurons and set the stage to further evaluate their role in the NF-1 pathophysiological process. Future studies will reveal whether human aging, polymorphisms or environmental factors alter the expression levels of these miRNA and, thus, contribute to variability in NF-1 symptoms.
